# Comparative Study of Epidemiological and Anthropological Aspects of Diabetes and Hypertension in Cameroon

**Published:** 2016-01-11

**Authors:** N Tsabang, E Fongnzossie, D Donfack, CG Yedjou, PB Tchounwou, JZ Minkande, C Nouedou, PD Van, Sonwa

**Affiliations:** 1 Center for Research on Medicinal Plants and Traditional Medicine, Institute of Medical Research and Medicinal Plants Studies, Ministry of Scientific Research and Innovation, Yaounde, Cameroon; 2 University of Douala, Cameroon; 3 Faculty of Education, Department of Specialized Education Sciences, Yaounde, Cameroon; 4 Cellomics and Toxicogenomics Research Laboratory, NIH-RCMI Center for Environmental Health, Jackson State University, Jackson, Mississippi, USA; 5 General Hospital of Yaounde, Cameroon and Faculty of Medicine and Biomedical Sciences, Cameroon; 6 Faculty of Science Engineering, Universitteit Ghent, Coupure Links 653B-9000 Ghent, Belgium; 7 Center for International Forestry Research (CIFOR), Yaounde, Cameroon

**Keywords:** Diabetes, Arterial hypertension, Indigenous people knowledge, Traditional health care improvement

## Abstract

The traditional medicine in Africa in general and specifically in Cameroon does not manage diabetes and arterial hypertension very well. Yet, these pathologies are becoming more prevalent among the populations that need adequate knowledge to fight against them. Therefore the present study was designed to determine the knowledge, attitudes and practices of indigenous people regarding diabetes and hypertension control, and to assess the epidemiological aspects of these diseases in order to reinforce their health education and promote a better health care through traditional medicine.

To achieve this objective, 1,131 households including 70 traditional healers, 114 diabetics, 167 hypertensive patients, 30 hypertensive patients-diabetics and other Cameroonians were questioned on their ethnomedical knowledge of diabetes and arterial hypertension. Fifty-eight randomly distributed tribes were taking in account. The elucidation of anthropological and epidemiological aspects of diabetes and hypertension improved the beliefs of indigenous people and facilitated the modernization of diabetes and hypertension comprehension that remained focused on the elucidation of diseases' causes and complications, as well as on the behaviors that could help translate biomedical terms into locally meaningful metaphors.

## Introduction

High blood pressure and high glycaemia are universally accepted as two of the most important risk factors in the development of cardiovascular disease and they still represent a serious public health problem worldwide [1]. Diabetes and hypertension have been for a long time considered to be more prevalent in developed countries. Nevertheless, 62% of people with diabetes are undiagnosed in Africa and 86% of known diabetics live in low and middle income countries [2,3]. Particularly in Cameroon, where epidemiological studies have shown that 10% of the population is diabetic, 90% of them are obese at the beginning of the disease. Among the Cameroonian patients, these pathologies are the foremost cause of extremity amputation and acquired renal diseases. They are also associated with an increased risk for ischemic heart disease and infections [4] and contribute to higher risk for depression [5]. In Cameroon, the prevalence of hypertension adjusted to the age is 16.6% in men and 12.6% in women in urban population [6]. While in Teaching Hospital of Brazzaville, Congo Democratic Republic, arterial hypertension constituted 5.1% among 825 admissions. Patients were divided in 4 groups: Chronic hypertension (n=4), pre-eclampsia presumed on chronic hypertension (n=4), pregnancy hypertension only (n=15), pre-eclampsia (n=19). Primary gravidity (38.1%) and family history of hypertension (40.4%) were the most frequent risk factors. Obesity, gemality, previous pre-eclampsia represented respectively 14.3%, 9.5% and 4.8% [7]. Arterial hypertension represents the cardio-vascular affection most frequent among the black population in Africa [8]. In this study the anthropological and the epidemiological aspects of diabetes and hypertension were determined and were addressed for promoting the indigenous people' knowledge reinforcement and their better health care through traditional medicine.

Cultural factors in the prevalence of diabetes and hypertension in Cameroon are mainly attributed to diet, although other relevant cultural characteristics include lifestyles, attitudes about disease prevention, and practices related to personal autonomy and giving up advice. In the African continent the cultural diversity is favorable to the use of alternative modes of health care including familial self-medication, traditional healers' treatments and market procurement of medicines. Hence, the following research questions were developed: Are the hypertensive patients and/or diabetics able to confront the anthropological aspects of these dangerous diseases with their epidemiological manifestations to have more useful understanding for high-quality of early treatments? In other words how to promote successful treatments of diabetics and/or hypertensive patients in the households and at the traditional healers? The search for potential answers to these research questions is the key determinant for the setup of the methodology.

## Methodology

### Condition of selection of patients

Patients admitted in this study have been diagnosed at least once in medical centers and confirmed by presenting a medical book or a bulletin of medical exam that certified their diabetic and/or hypertensive state at the moment of their selection.

### Anthropological aspects

The survey of the beliefs of indigenous Cameroonians regarding diabetes and arterial hypertension diseases was carried out on 1131 households that included 114 diabetics, 167 hypertensive patients, and 30 hypertensive patients with diabetes. These patients respectively represented 10.08%; 14.76% and 2.65% of respondents, and all together constituted 27.50% of the total interviewees. Fifty-eight socio-cultural groups were taking in account, in a random distribution in Cameroon. These respondents provided information on their cultural and ethno medical knowledge on diabetes and arterial hypertension. The following questions were addressed to each of them: Do you know about diabetes and arterial hypertension diseases? If yes, how do you diagnose them? What do you think about these pathologies? Are you a diabetic and/or hypertensive patient? Is there someone in the family who is a diabetic and/or hypertensive patient? Are there myths, tales or legends around these diseases?

### Representativeness of recorded respondents

From a sample of 1131 households that included 328 diabetics and hypertensive patients, data on the beliefs of Cameroonians were collected on diabetes and hypertension. Fifty percent of respondents were males and 50% were females. From the first nine hundred and twenty-three respondents all the data were recorded. Therefore from the 208 remaining respondents, no new data were registered. So the number of 1131 interviewees was representative of Cameroon population. The answers of the following questions permitted to establish the representativeness of recorded types of patients using statistical analysis. Are there significant differences between the recorded types of diabetics, of hypertensive patients and of hypertensive patients-diabetics when compared two by two? In the other words: Do the recorded types of patients (diabetics, hypertensive patients and diabetics-hypertensive patients) present the same prevalence in Cameroon' population, if in the same condition the survey is repeated?

### Statistical analysis

Study participants were categorized in different subgroups including: type 1 diabetes, type 2 diabetes, essential hypertension and secondary hypertension, diabetes with essential hypertension, diabetes with secondary hypertension, males, and females. From the data generated from these groups, statistical analyses were performed to compare the disease prevalence rates between specific paired groups including type 1 diabetes and type 2 diabetes; essential hypertension and secondary hypertension; diabetes-hypertension with essential and with secondary hypertension, and male and female patients. Statistical differences were assessed using standard protocol as described in “Statistical Methods in Biology” [9].

## Results

### Number of recorded patients

Three hundred and twenty-eight (328) patients were recorded and divided into groups: clinical followed up patients and not clinical followed up patients (Table 1). The first group used medicinal plants in familiar herbal remedies and the second did not use.

### Epidemiological aspects

#### Prevalence comparison of type 1 diabetics (insulin-dependent diabetics) and type 2 diabetics (non- insulin-dependent diabetics)

The number of insulin-dependent diabetics and non-insulin-dependent diabetics recorded was respectively 39 and 85. If the two types of diabetes have the same chance to be met during the survey, there is equal probability: p=q=½. The two types of diabetes form each a binomial distribution. It was about to compare the observed percentage for each type of diabetes to hypothetic value. According to the hypothesis zero (no difference between the two types of diabetes) the two types of diabetes have the same chance to appear during the survey. In this case they have the same prevalence in the population. The variance of this probability is

V=pq/n with n=124

V=1/2 × 1/2/124=0. 0020161. Standard error:=0.044901325.

The observed values were:

From IDD sample:

a/n ± =0. 6855 ± 0. 0416

a=85.

From NIDD sample:

a/n ± =0, 3145 ± 0, 0416

a=39

The different observed gap of hypothetic value is:

From NIDD sample: 0.6855–0, 5000=0.1855;

From IDD sample: 0.5000–0.3145=0.1855.

By definition, this gap is 2.6 times superior to standard error; we concluded that there was a significant difference between these 2 types of diabetes at 95%. IDD was dominant. Until what limits the variations of each disease can be put on the account of chance fluctuations? To answer this question, the confidence interval of diabetes was determined.

The normal variable with zero mean or the unit standard deviation was

$$d=a/n-p:\sqrt{p(1-p)/n}=4.131.$$

The confidence intervals for the true unknown proportion were: 80.869 and 89.131 for NIDD: and 34.869 and 43,131 for IDD at 95% coefficient of security. The probability so that the observed proportions of diabetic's types remained in these intervals, if the survey began again in the same conditions, was more increased. This sample was widely representative for the two types of diabetes in Cameroonian's population.

#### Prevalence' comparison of hypertensive patients with essential and secondary hypertensive patients

The similar analysis previously realized has permitted to demonstrate the non-existence of a significant difference between essential hypertension and secondary hypertension, because the observed gap of hypothetic value (0.0928) was < Standard error (0.03869 × 2.6).

#### Prevalence' comparison of diabetics with essential and diabetics with secondary hypertension

In the same condition the similar analysis previously realized has permitted to demonstrate the existence of a non-significant difference between diabetics with essential hypertension and diabetics with secondary hypertension. The observed gap of hypothetic value (0.1756) was < Standard error (0.038219 × 2.6).

### Sex distribution of recorded patients

The similar analysis previously realized has permitted to demonstrate the nonexistence of a significant difference between type 2 male diabetics and type 2 female diabetics. The observed gap of hypothetic value (0.0882) was < Standard error (0.0542) × 2.6. Also between male secondary hypertensive patients and female secondary hypertensive patients there wasn't a significant difference. The observed gap of hypothetic value was (0.1323) < Standard error (0.060632 × 2.6). Nevertheless, there is a significant difference between male essential hypertensive patients and female essential hypertensive patients. The observed gap of hypothetic value (0.1969) was > Standard error (0.03869 × 2.6). The normal variable with zero mean or the unit standard deviation was d=a/n–p: =0.1972. The confidence intervals were: 29.802 and 31.1972 for essential male hypertensive patients and 68.802 and 69.1972 for female essential hypertensive patients at 95% coefficient of security (Table 2). The probability so that the observed proportions of male essential hypertensive patients remained in these intervals, when we began again the survey in the same conditions was more increased. This sample was widely representative for the proportions of men diabetics and women diabetics, in Cameroonian's population.

### Anthropological aspects

According to the respondents, the anthropological aspects described below were raised from the beliefs of population. Indeed 62% of respondents considered diabetes and arterial hypertension as being two mystic diseases. They believed that the coma (Menoubia), the numbness (Aloute me kouc), the dermal clues (Emock Dem), the gangrenes (Ta-Nteu Nfeung), the retinopathy (Meneck Se'eung), the nephropathy (Letse kekan) and the cardiac complications like arteriosclerosis (Ate'eu ngan net) and the grave noose bleeding (Ta-Nteu Afifi), were linked to the suffering of patients' totems, killed by the traditional sporting-gun or victim of a grave fall in the invisible world. The above vernacular names in brackets were the complications' designations in Bamileke. At these state of diseases, the patients were usually considered as sorcerers and the complications they developed came from their expeditions in the invisible world. The patients could also suffer from severe attacks of witchcraft, the punishments of ancestors, the maledictions or the false gods. Then these false gods must be appeased at all costs. Likewise, the gangrenes of the foot and the benumbed inferior members came from the stampings of conspicuous or invisible poisons. The paralysis and the hemiplegia attacked bewitched patients.

The survey suggested that 10% of 70 traditional healers, living in the hinterland were illiterate; therefore they diagnosed diabetes by the color (clear or dim) of urine, the strong density of insects (flies, bees and ants) around it on the soil surface, and also by its sweet taste. It is not recommended to taste the urines because it is rich in toxic substances such as uric acid and by metabolic products. Only traditional healers are able to make a good traditional diagnosis and cure of victims. The healings were researched during the nocturne sessions of treatment of mystic, the ceremonies of healing bewitched patients, the cults to ancestors, the gift of foods, drinks, clothes, and/or money to family or clan members, and the sacrifices to idols.

The restitution of the present study, three years ago, has changed gradually the behavior of indigenous Cameroonians. Many traditional healers, especially those of hinterland produce less and less the incantations and the speculations that retarded the health care of diabetics and/or hypertensive patients in the hospital centers. The good understanding of diabetes and hypertension epidemiology and manifestations including progression and complications, can save the broken relationships between familial' members due to the consequences of the consultation of native doctors. Nevertheless, the diagnostic of traditional healers, for those who effusively believed them, reduced the stress of patients and enhanced their health care.

### Manifestations and difficulties of health care

Far to claim a pejorative judgment for these indigenous considerations, diabetes and hypertension are insidious diseases (hidden beginning and slow evolution), with detection often late and with sudden release. There are chronic pathologies with generally long and expensive treatments. The monthly costs for treating each diabetic, hypertensive, and diabetic-hypertensive patient are respectively 22,500 Fcfa, 22,500 Fcfa and 45,000 Fcfa in the case of uncomplicated diabetes and hypertension. The cost of diet increases the financial expenses. Certain combinations of antidiabetic or antihypertensive treatments, composed from the following products: insulatard HM (14,525 Fcfa), Actrapid HM (14,510 Fcfa) like insulins, Hexen 50 (13,860 Fcfa), Lodoz (10,415 Fcfa) like hypotensive products and Glucophage 850 mg (9,190 Fcfa) representative of oral hypoglycemic products, are rare in rural or onerous for patients. Then the difficulties of access to the medicines can facilitate the appearance of the following severe complications: coma, numbness of members and muscle atrophy, on diabetics; hypertrophy of left ventricle, occlusion of heart artery (infratus) and of brain artery (cerebral softening), vascular-cerebral accidents (AVC), on patients; gangrenes (local necrosis of a tissue), nephropathy (deficiency or renal insufficiency), on diabetics and/or hypertensive patients, rendering the treatment difficult and prohibitive. The patients are terminally sick and face access restriction to modern medicines. Therefore 76% of recorded patients live with families who are financially deprived, have limited resources, and rely on alternative modes of treatment over modern medicine. However, the 24% of patients who are financially sufficient also rely on medicinal plants for a total recovery because of the chronic character of the two diseases that makes the modern treatment very lengthy.

The ethnopharmacological preparations of the plants used were previously described by Tsabang [10]. Plants are potential sources for medicinal treatment because the rural population, mostly of the hinterland and far from urban zones well supplied with manufactured pharmaceutical products, have developed important experiences on the use of medicinal plants and natural products.

## Discussions

There was a statistically significant difference (p<0.05) between the proportion of type 1 diabetics and that of type 2 diabetics. Hence, the two forms of diabetes did not affect the young population (0 to 30 years) and the adult population (more than 30 years) at the same prevalence rate. It has been reported that most diabetics eat much food and this favors obesity near 45 years [11]. Thirty percent of obese persons become type 2 diabetics [12]. In Cameroon, 90% of population is type 2 diabetic [13]. This is the confirmation of why in our sample; the number of type 2 diabetics was dominant. The comparison between essential hypertensive patients and secondary hypertensive patients revealed a non-significant difference in disease rate between these two types of hypertensive patients. Yet, essential hypertension (also called primary hypertension or idiopathic hypertension) is the most common type of hypertension, affecting 95% of hypertensive patients [14,15]. Prevalence of essential hypertension increases with age, and individuals with relatively high blood pressure at younger ages are at increased risk for the subsequent development of hypertension. The observed variations of each type of hypertension can be attributed to fluctuations in specific risk factors. Hypertension can increase the risk of cerebral, cardiac, and renal complications [16].

There was no significant difference between diabetics and/or hypertensive male (50%) and female (50%) patients. Previous works presented below show that this tendency is observed elsewhere in the study of the prevalence of hypertension and diabetes. The age-adjusted prevalence of hypertension was 34%, 25.4%, and 23.2% for men and 31%, 21%, and 21.6% for women among blacks, whites, and Mexican Americans, respectively. NHANES III reported the prevalence of hypertension was 12% for white men and 5% for white women aged 18-49 years. However, the age-related blood pressure rise for women exceeded that of men. The prevalence of hypertension was reported at 50% for white men and 55% for white women aged 70 years or older [17]. The prevalence of diabetes was 5.7% (95% confidence interval-CI: 4.8-6.8) overall, with a slight difference between the men (5.5%; 95% CI: 4.1-7.2) and women (5.9%; 95% CI: 4.6-7.6). The prevalence of diabetes among men was not significantly different from that among women (Owner's Risk: 1.01; 95% CI: 0.91-1.11) [18].

There is a significant difference between male essential hypertensive patients and female essential hypertensive patients, in favor of women who are fatter than men. The relation of hypertension with obesity is well established. The reduction of body mass index (BMI) to values less than 23 would prevent 46.7% of future hypertensive cases in men. Hypertension increases progressively with age in both genders, although the prevalence is higher for men than for women (45.2% vs 28.9%) in all age groups [1].

Diabetes and hypertension are the exceptionally difficult diseases to manage. For the individual, it requires a lifelong commitment to dietary change, physical exercise, and self-monitoring of a medication regime (oral or injectable products) and lastly dialysis. Diabetes and hypertension health care requires the work of a team of health providers-internal medicine or family medicine practitioners, diabetes and hypertension educators, nutritionists, podiatrists, and dentists-ideally in regular communication with each other [19].

## Conclusion

This study reveals that diabetes and hypertension are increasingly prevalent in the population of Cameroon. It is also divulges that many factors including the anthropological considerations, the increasing number of diabetics and hypertensive patients, the chronic nature of diabetes and arterial hypertension, and the expensive costs of modern treatments, facilitate the recourse to traditional medicine. Because of their poor financial conditions, the patients living in affected families look for alternative modes of therapy instead of physicians' treatment using modern medicine [20]. Collaborations with physicians are needed for a better diagnostics and a good health care of patients. Nevertheless the mystic considerations are harmful for the familial' cohesion in Cameroon and retard the medical care of patients. However, they present some good cultural aspects that include stress reduction due to the self-confidence of patients, because of the appeasement of false gods, of bewitches and diverse aspects of witchcraft. For rapid diagnosis, we recommend patients to consult physicians first and to use medicinal plants in collaboration with physicians. We also recommend traditional healers to avoid the diagnosis of diabetes by tasting the urines rich in toxic substances like uric acid [21]. In general four domains (political, economic, biological/genetics, and socio-cultural) influence diabetes and hypertension management. Each of these domains should be reflected in program models and tribal policies for the control/prevention of diabetes and hypertension in Africa.

## Figures and Tables

**Table 1:** Distribution of diabetics and/or hypertensive patients recorded.

Patients	Diabetics		Hypertensive patients		Diabetic-hypertensive patients	
	NIDD	IDD	ETH	SHT	NIDD-SHT	NIDD- EHT
**Clinically followed up patients**						
**Men**	30	15	4	20	03	01
**Women**	40	21	6	36	04	02
**Total**	**70**	**36**	**10**	**56**	**07**	**03**
**Not clinically followed up patients**						
**Men**	05	3	15	5	6	2
**Women**	10	0	24	7	12	7
**Total**	15	3	89	12	18	9
**Total per type of diseases**	85	39	99	68	25	12
**Total per diseases**	124		167		37	

NIDD: Non Insulin-Dependent Diabetes; IDD: Insulin-Dependent Diabetes; EHT: Essential Hypertension; SHT: Secondary Hypertension.

**Table 2:** Sex distribution of diabetics and/or hypertensive patients recorded.

Patients	Diabetics		Hypertensive Patients		Diabetics-hypertensive patients	
	NIDD	IDD	ETH	SHT	NIDD-SHT	NIDD- EHT
	**Men**					
	35	18	30	25	9	3
	**Women**					
	50	21	69	43	16	9
**Total**	**85**	**39**	**99**	**68**	**25**	**12**

NIDD: Non Insulin-Dependent Diabetes; IDD: Insulin-Dependent Diabetes; EHT: Essential Hypertension; SHT: Secondary Hypertension.
